# Signaling pathways and ion channels in osteoarthritis: a review of recent advances

**DOI:** 10.3389/fimmu.2026.1823905

**Published:** 2026-08-06

**Authors:** Xi Liu, Niqin Xiao, Qiumei He, Kuanmeng Chi, Hongting Lu, Qianqian Yang, Jian Wang, Zhaohu Xie, Zhaofu Li

**Affiliations:** Yunnan University of Chinese Medicine Kunming, Yunnan, China

**Keywords:** osteoarthritis, pathogenesis, signaling pathways, ion channels, inflammation

## Abstract

The understanding of osteoarthritis (OA) has gradually evolved from the traditional perspective of it being a mere “wear-and-tear” joint disease to a more comprehensive view that characterizes it as a degenerative joint disease marked by chronic low-grade inflammation and immune imbalance. The progression of OA is closely linked to abnormal mechanical loading and the activation of the innate immune system. This narrative review examines the role of mechanosensory-immune signaling networks in OA, with a specific focus on ion channels as receptors for upstream mechanical and chemical stimuli. These channels facilitate signal transduction through ion fluxes, such as Ca²^+^, to key inflammatory pathways, including nuclear factor κB (NF-κB), cyclic guanosine monophosphate-adenylate synthase-interferon gene stimulator (cGAS-STING), adenylate-activated protein kinase (AMPK), and the NLRP3 inflammasome. Furthermore, it integrates signaling axes such as the protein kinase/Yes-related protein (Hippo/YAP) pathway, the Wnt/β-catenin pathway, and the phosphoinositide 3-kinase/protein kinase B/mammalian target of rapamycin (PI3K/AKT/mTOR) signaling axes, analyzing their context-dependent bidirectional regulation during cartilage homeostasis maintenance and the degeneration process. The primary contributions of this study include the construction of an integrated mechanobiological-immunological transduction network framework that encompasses “ion channels–signaling pathways–immune responses.” This framework highlights the hierarchical crosstalk and positive feedback amplification effects among these pathways, while exploring their dynamic and reversible regulatory characteristics under varying mechanical and inflammatory microenvironments. Ultimately, this framework facilitates a systems-level understanding of the pathological progression of OA and provides a theoretical foundation for the development of multi-target synergistic therapies and stage-specific precision intervention strategies.

## 1 Introduction

Osteoarthritis (OA) is a disease that affects the entire joint, characterized by primary pathological features such as degeneration of articular cartilage, subchondral bone remodeling, synovial inflammation, and osteophyte formation. The pathogenesis of OA involves complex interactions between abnormal mechanical loading and immune-inflammatory responses. Recent studies indicate that osteoarthritis is not simply a ‘wear-and-tear’ condition; rather, it is an immune-related disease primarily defined by persistent, low-grade chronic inflammation and abnormal activation of innate immune signaling pathways (1, 2). OA predominantly involves weight-bearing joints or those with high mobility, such as the hands, knees, and hips. Clinically, the main symptoms include joint pain, swelling, morning stiffness, and bony overgrowth. In advanced stages, joint deformities may arise, leading to significant impairment of joint function. At the microscopic level, OA is characterized by cartilage fissuring and erosion, synovitis, and abnormal bone formation (3, 4). Imaging and histopathological studies have demonstrated that synovitis is prevalent in OA. Magnetic resonance imaging (MRI) findings indicate that approximately 60% of OA patients exhibit synovial inflammation or hypertrophy. Furthermore, the severity of synovitis is significantly correlated with the progression of cartilage damage and the experience of joint pain (5, 6). The immune cells infiltrating the synovial tissue are predominantly macrophages, which account for approximately 65% of the total infiltrating cell population. The inflammatory cytokines released by M1-polarized macrophages—including TNF-α, IL-1β, and IL-6—establish a cytokine network that drives the degradation of the cartilage matrix (7). The CCL2/CCR2 chemokine axis is crucial in mediating the directed recruitment of monocytes and macrophages to the synovium; its activation not only promotes the persistence of synovial inflammation but is also closely associated with the severity of OA pain (8). Therefore, conducting a systematic immunological analysis of the pathogenesis of OA holds significant importance. Currently, OA is recognized as a leading cause of disability in China. Its prevalence and progression are correlated with factors such as age, gender, body weight, educational level, and socioeconomic status (9). Research indicates that the incidence of OA is strongly associated with advancing age. With the acceleration of global aging trends, data released by the World Health Organization show that approximately 528 million people worldwide were affected by OA in 2019, about 73% of whom were aged 55 years or older (10). According to a recent study published in *The Lancet Rheumatology*, it is projected that nearly one billion individuals worldwide will be affected by OA by 2050 (11). Cartilage is a highly differentiated tissue characterized by the absence of blood vessels, nerves, and lymphatic vessels (12). Consequently, once damaged, it is challenging to regenerate. Currently, treatment options for OA remain limited, and there is no definitive method to cure affected patients (9). The primary treatment approach consists of conservative measures, including anti-inflammatory and analgesic therapies, as well as chondroprotective therapy. When the disease progresses to an advanced stage, surgical interventions, such as joint replacement, are typically employed. However, the core objective of these treatment approaches primarily focuses on controlling pain and inflammatory responses (13, 14). Therefore, an in-depth analysis of the mechanisms underlying innate immune activation in OA, the regulation of synovial macrophage polarization, and the cascade amplification of inflammatory signaling networks is of great significance for identifying new immunological therapeutic targets. This article reviews recent advances in the study of the pathogenesis of OA, focusing on the integrative roles of signaling pathways and ion channels in the mechanostatic-inflammatory-immune transduction network, and discusses the limitations of current research and future directions, with the aim of providing guidance for immunological research on OA.

## 2 Signal pathways

The pathogenesis of (OA is highly intricate, primarily characterized by chondrocyte apoptosis and reduced cellular activity. These cellular behaviors are critically influenced by mechanical signals within the cellular microenvironment (15). Current evidence suggests that multiple signaling pathways play regulatory roles in the progression of OA. Key pathways involved include the Hippo/Yes-associated protein (Hippo/YAP), Wnt/β-catenin, nuclear factor kappa B (NF-κB), adenosine monophosphate-activated protein kinase (AMPK), cyclic GMP–AMP synthase–stimulator of interferon genes (cGAS–STING), and phosphoinositide 3-kinase/protein kinase B/mammalian target of rapamycin (PI3K/AKT/mTOR) signaling pathways. The following discussion will focus on the role of these six signaling pathways in the pathogenesis of OA, with the aim of providing insights for identifying additional therapeutic targets for OA.

### 2.1 Signaling pathways involved in mechanical responses: Hippo/YAP and Wnt/β-catenin signaling pathways

#### 2.1.1 Hippo/YAP signaling pathway

First identified in *Drosophila*, the Hippo signaling pathway is a highly evolutionarily conserved key regulator of organ size. The Hippo signaling pathway is a crucial regulator of tissue development and homeostasis. Vanyai et al. (2020) demonstrated in a mouse embryonic cartilage development model that the Hippo-YAP pathway influences chondrogenesis by modulating the expression of connective tissue growth factor (Ctgf), cysteine-rich protein (Cyr61), and various matrix-remodeling enzymes. This modulation impacts chondrogenesis and suggests that the pathway may play a role in how chondrocytes perceive and respond to mechanical signals from the extracellular matrix (16).The core of the Hippo signaling pathway comprises a kinase cascade, along with the involvement of transcriptional coactivators YAP and TAZ, as well as DNA-binding transcription factors (17). The Hippo/YAP signaling pathway is intrinsically linked to tissue regenerative capacity and primarily regulates cellular morphogenesis. This pathway functions as a mechanosensor in mammals. The Hippo/YAP signaling pathway serves as a mechanosensor in mammals. Zhong et al. (2013) demonstrated *in vitro* that the stiffness of the extracellular matrix (ECM) can influence the subcellular localization of YAP in terminally differentiated chondrocytes. Downregulation of YAP expression contributes to the maintenance of the chondrocyte phenotype and inhibits proliferation. Conversely, inactivation of the LATS1 kinase promotes chondrocyte dedifferentiation specifically on a stiff matrix (18). Maintaining cartilage homeostasis is essential for preventing the onset and progression of OA. The Hippo/YAP signaling pathway significantly contributes to this process. In a chondrocyte microenvironment characterized by low levels of reactive oxygen species (ROS), chondrocytes can interact with the extracellular matrix (ECM) and activate YAP in a hypoxia-inducible factor-1 (HIF-1)α-dependent manner, thereby preserving the stability of cartilage morphology (19, 20). The differentiation process of chondrocytes is regulated by mechanical stress. Such stress modulates YAP expression, promoting the activation of chondrogenic marker proteins and facilitating YAP nuclear translocation. This process further influences the transcriptional regulation of proteasomal degradation, thereby playing a key role in chondrocyte development, differentiation, and the maintenance of cartilage phenotypic stability. Ultimately, it contributes to accelerated cartilage repair and the maintenance of joint metabolic homeostasis (15, 21, 22). TheECM, with appropriate physiological stiffness, facilitates the cytoplasmic retention of YAP through mechanosensitive mechanisms in response to mechanical stress, thereby contributing to the maintenance of chondrocyte phenotypic stability (15, 23). Sladitschek-Martens et al. (2022) demonstrated in various mouse stromal cell models that the maintenance of YAP/TAZ mechanotransduction activity inhibits cGAS-STING signaling, thereby preventing cellular senescence associated with a reduction in YAP/TEA activity (24). YAP binds to TEA domain transcription factors (TEADs) to activate expression of the senescence-protective protein forkhead box D1 (FOXD1), thereby delaying the progression of OA (25). In a study by Deng et al. (2018) (26), it was found that YAP-overexpressing mice exhibited downregulation of MMP13 expression in articular cartilage, an increase in the expression of Collagen Type II Alpha 1 Chain (COL2A1), a reduction in the number of aged mesenchymal stem cells (MSCs), inactivation of the NF-κB signaling pathway, and a delay in bone erosion. Furthermore, upon binding to the transcription-enhancing domain (Teads), YAP1 directly promotes the expression of its downstream target SOX6, thereby facilitating the proliferation of early-stage chondrocytes (27). In a mouse model featuring chondrocyte-specific Runx1 knockout, the overexpression of Runx1 significantly elevates YAP expression in chondrocytes, effectively integrating the Wnt/β-catenin signaling pathway to sustain cartilage homeostasis. Conversely, Runx1 deficiency leads to a reduction in YAP levels, accompanied by an increase in matrix metalloproteinase 13 (MMP-13) and a disintegrin and metalloproteinase with thrombospondin motifs 5 (ADAMTS-5) (28).YAP interacts with the DNA-binding transcription factor Runx2 to inhibit the production of the target gene X-type collagen alpha 1 chain (COL10A1), thereby suppressing abnormal chondrocyte hypertrophy and maintaining cartilage homeostasis (27).

The Hippo/YAP pathway is intricately involved in OA pathogenesis, influencing its development and progression through diverse aspects. Current research has not yet fully elucidated its mechanisms, as it appears to exert both protective and detrimental effects across different stages and contexts of OA (15). Under excessive mechanical stress, YAP translocates to the nucleus and binds to Tead transcription factors, promoting the increased expression of Runx2. This, in turn, stimulates the production of COL10A1, further enhancing bone hypertrophy and calcification, thereby contributing to OA. *In vitro* studies have demonstrated that physiological stimuli, such as 400 mOsm osmolarity or IGF-1, promote the nuclear translocation of YAP by inducing phosphorylation at Ser128. This process enhances SOX9-dependent COL2A1 enhancer activity, resulting in the upregulation of COL2A1 and aggrecan (ACAN) expression, and exerts a chondroprotective effect (Cui et al., 2023) (29). However, in a DMM-induced mouse model of OA, it was observed that overexpression of nuclear YAP leads to the downregulation of COL2A1 and SOX9, thereby accelerating cartilage degeneration (, 2020) (23). In the context of chondrocyte proliferation and apoptosis, research involving the murine chondrogenic cell line ATDC5 and a mouse model of OA indicates that YAP overexpression inhibits the proliferation of ATDC5 chondrocytes. This overexpression concurrently suppresses the ubiquitination of beclin-1, which leads to the inhibition of chondrocyte autophagy, promotes chondrocyte apoptosis, and contributes to the progression of OA (Zhang et al., 2019) (30). Subchondral bone remodeling, destruction of articular cartilage, and calcification at the osteochondral junction are widely recognized as synchronous processes during the progression of OA (31). The Hippo/YAP signaling pathway also plays a role in subchondral bone remodeling and has been shown to exert a protective effect in OA. Specifically, in a mouse model of destabilization of the medial meniscus (DMM)-induced OA, the knockdown of YAP resulted in impaired subchondral bone formation (32).

#### 2.1.2 Wnt/β-catenin signaling pathway

In addition to its functions in embryonic development—where it governs cell development, differentiation, and polarization—the Wnt pathway is critically important for the maintenance of adult cartilage homeostasis. This pathway functions primarily by promoting chondrocyte survival and inhibiting their hypertrophy (33). β-Catenin serves as a core effector of the canonical Wnt/β-catenin signaling pathway (34). Wnt signaling is broadly categorized into two types: the canonical pathway, which is Wnt ligand-dependent and relies on β-catenin-mediated transcription, and the non-canonical pathway, which operates independently of β-catenin (35). Upon binding of Wnt ligands, the Wnt signaling pathway facilitates the nuclear translocation of β-catenin, resulting in its accumulation within the nucleus. Subsequently, β-catenin associates with transcription factors T cell factor/lymphoid enhancer factor (Tcf/Lef) to form a transcriptional complex. This complex further recruits the YAP/TAZ/Tead co-activator complex, synergistically activating downstream gene expression—including upregulation of matrix metalloproteinases (MMPs) (36, 37). The accumulation of MMPs in chondrocytes contributes to the destruction of these cells. Concurrently, activation of the Wnt pathway induces the expression of downstream Wnt1- inducible signaling pathway protein 1 (WISP-1), which in turn stimulates chondrocytes to secrete metalloproteinases, including MMPs and ADAMTS, leading to cartilage matrix degradation (38). In the non-canonical Wnt pathway, Frizzled binds Wnt ligands and activates co-receptors such as protein kinase C (PKC) and c-Jun N-terminal kinase (JNK), leading to the upregulation of downstream target genes and activation of calcium-related pathways that contribute to OA pathogenesis. However, the precise mechanisms involved remain to be fully elucidated (35).

### 2.2 Key inflammatory pathways: NF-κB and cGAS-STING signaling pathways

#### 2.2.1 NF-κB signaling pathway

OA is a multifaceted joint disorder characterized by progressive erosion of articular cartilage, sclerosis of the subchondral bone, osteophyte formation, and chronic synovitis. Although the precise mechanisms driving OA remain incompletely understood, elevated levels of pro-inflammatory cytokines and matrix metalloproteinases have been consistently detected in both the synovium and articular cartilage of OA patients (39). Activation of the NF-κB signaling pathway further supports this observation. NF-κB represents a critical transcription factor in mammals, with its key subunits including RelA (p65), RelB, c-Rel, as well as the precursor proteins NF-κB1 (p50) and NF-κB2 (40). This pathway plays an essential role in regulating cell cycle progression, ECM degradation, apoptosis, and inflammatory responses (41). Its canonical activation is primarily triggered by pro-inflammatory cytokines, especially tumor necrosis factor α (TNFα), which bind to their corresponding receptors and initiate downstream signaling leading to the activation of transcription factors RelA/p65 and p50. This mechanism primarily depends on the catalytic functions of IKKβ and IKKγ subunits within the IκB kinase (IKK) complex. Upon activation, IKKβ catalyzes the phosphorylation of the inhibitory protein IκBα, thereby marking it for ubiquitination and subsequent degradation via the proteasome pathway. This results in the release and subsequent nuclear translocation of the RelA-p50 heterodimer, enabling the transcription of target genes (40, 42). Upon translocation into the nucleus, the NF-κB complex—particularly the p65/p50 heterodimer—regulates the expression of target genes involved in critical biological processes such as cell proliferation, apoptosis, oxidative stress, and inflammatory responses. Specifically, after nuclear entry, p65/p50 binds to promoter regions of pro-inflammatory cytokine genes to enhance their transcription (43). The expression of nitric oxide (NO), inducible nitric oxide synthase (iNOS), and prostaglandin E_2_ (PGE_2_) is stimulated by key pro-inflammatory cytokines such as interleukin-1β (IL-1β) and interleukin-6 (IL-6). This cascade of reactions ultimately promotes the onset and progression of joint pain (44). This pathway induces the expression of hypoxia-inducible factor 2α (HIF-2α) and transcription factors such as RUNX2, which in turn stimulate the production of MMPs and ADAMTS (35), leading to degradation and degeneration of articular cartilage. Furthermore, the activation of the NF-κB pathway upregulates the expression of NOD-like receptor family pyrin domain-containing 3 (NLRP3), as well as pro-IL-1β and pro-IL-18, providing the necessary molecular basis for the assembly of the NLRP3 inflammasome (45, 46). In the presence of reactive oxygen species (ROS) and potassium ion (K+) efflux, the NLRP3 inflammasome assembles and activates Caspase-1, which cleaves inactive pro-IL-1β into mature IL-1β and induces pyroptotic cell death (45). The expression of cytokines and chemokines, in turn, activates the NF-κB signaling pathway via a positive feedback loop, leading to a vicious cycle that exacerbates OA (47). The NF-κB pathway can be activated by inflammatory mediators, mediating chronic inflammatory responses and influencing the development of autoimmune diseases, making it a crucial component in the progression of OA (41). In experimental mouse models of OA and *in vitro* chondrocyte cultures, Deng et al. (2018) demonstrated that the crosstalk between the NF-κB and Hippo-YAP signaling pathways plays a crucial role in mediating the pathogenesis of OA. Furthermore, they revealed that these two pathways exert antagonistic effects on each other, specifically, inflammatory cytokines induce phosphorylation of transforming growth factor-β-activated kinase 1 (TAK1), which facilitates the ubiquitin-mediated degradation of YAP/TAZ. The subsequent reduction in YAP/TAZ protein levels enhances the availability of substrates for TAK1, leading to further activation of the NF-κB pathway. This reciprocal regulation establishes a pro-inflammatory feedback loop that exacerbates OA progression (15, 26).

#### 2.2.2 cGAS-STING signaling pathway

The cGAS–STING pathway, a key cytoplasmic DNA-sensing mechanism, plays an important role in recognizing endogenous DNA under various pathological conditions such as autoimmune diseases and cancer (48). Furthermore, they are implicated in the regulation of OA development. This signaling axis centers on two core components: cyclic GMP–AMP synthase (cGAS) and stimulator of interferon genes (STING) (49). Under inflammatory conditions, chondrocytes subjected to cellular stress and damage release mitochondrial DNA (mtDNA) into the cytoplasm, which subsequently activates the cGAS–STING signaling pathway (50). The cGAS–STING pathway mediates OA pathogenesis through its downstream effectors, including interferon regulatory factor 3 (IRF3), TANK-binding kinase 1 (TBK1), and IKK (49). Endoplasmic reticulum stress (ERS)induces STING activation through mitochondrial damage mediated by calcium ions and reactive oxygen species (ROS), resulting in DNA release. This process subsequently promotes the phosphorylation of IRF3 and IKKs. Following phosphorylation, these transcription factors translocate to the nucleus and enhance the expression of type I interferons and pro-inflammatory cytokines (48, 51, 52). It is noteworthy that STING upregulates inflammatory cytokines via the NF-κB signaling pathway (53) and increases the expression of NLRP3 and the interleukin precursor pro-IL-1β (54). In the context of mtDNA release induced by STING activation, the NLRP3 inflammasome is assembled and subsequently activates caspase-1, thereby triggering Gasdermin D (GSDMD)-mediated pyroptosis (53, 55). In addition, caspase-1 cleaves pro-IL-1β into its mature form, interleukin-1β (IL-1β), which further activates NF-κB signaling and amplifies the inflammatory response (56). Meanwhile, pyroptosis leads to the release of large amounts of mtDNA, which in turn activates the cGAS–STING pathway, thereby establishing a positive feedback amplification loop. Furthermore, The 29 kDa fibronectin N-terminal fragment (29 kDa FN-f), a large glycoprotein involved in the chondrocyte extracellular matrix (ECM), induces DNA damage in chondrocytes and activates the cGAS and STING pathways, leading to the expression of inflammatory cytokines. These effects are mediated through the Toll-like receptor-2 (TLR-2) and nucleotide-binding and oligomerization domain protein 2 (NOD2) signaling pathways. Silencing of TLR-2 or NOD2 inhibits the activation of the cGAS/STING pathway induced by 29-kDa FN-f (57). Overexpression of STING promotes degradation of the chondrocyte ECM by upregulating MMPs through the NF-κB pathway. Additionally, STING contributes to OA progression by inducing chondrocyte apoptosis and senescence. Conversely, inhibition of TBK1 expression protects against ECM degradation by suppressing cGAS–STING signaling. IKK, particularly IKKα, promotes monocyte chemotaxis and induces chondrocyte hypertrophy, thereby accelerating OA progression (49).

### 2.3 Signaling pathways involved in metabolic homeostasis: AMPK/SIRT and PI3K/Akt/mTOR signaling pathways

#### 2.3.1AMPK/SIRT signaling pathway

AMP-activated protein kinase (AMPK) is a serine/threonine protein kinase widely distributed in eukaryotes, activated under hypoxic stress to maintain intracellular energy homeostasis (58, 59). AMPK coordinates multiple pathways to maintain cellular energy homeostasis (60) and participates in mitochondrial quality control (MQC) within chondrocytes. MQC dysregulation is regulated by both the chondroprotective molecule AMPK and SIRT3. AMPK participates in mitochondrial damage processes, and its abnormal activity is closely associated with mitochondrial dysfunction and impaired mitochondrial biogenesis in chondrocytes, thereby mediating OA progression (35, 61). Reduced AMPK activity activates oxidative stress (OS), NF-κB, and pro-inflammatory cytokine production, inducing degenerative destruction of articular cartilage; SIRT enzymes, the most crucial deacetylases in mitochondria, maintain redox homeostasis to prevent oxidative stress in cellular metabolism through their activity linked to the binding of the conserved nicotinamide adenine dinucleotide (NAD+) (59, 62). Among these, SIRT3 is the most crucial mitochondrial deacetylase. Its activation deacetylates liver kinase B1 (LKB1), enhancing LKB1 activity to activate AMPK. Both AMPK and SIRT3 play regulatory roles in chondrocyte mitochondrial homeostasis (62), and they also mutually regulate each other, maintaining consistent expression and activity (59). AMPK senses energy changes in mitochondrial biogenesis. When cellular energy is depleted, AMPK is activated through phosphorylation to promote ATP production. Increased NAD+ levels activate SIRT3, which suppresses mutations in pre-existing mtDNA within chondrocytes of OA patients (59, 63), thereby delaying mitochondrial dysfunction and mitigating cartilage degradation in OA. AMP-activated protein kinase (AMPK)–sirtuin 3 (SIRT3)–pyruvate formate-lyase (PFL) axis (AMPK-SIRT3 PFL), mediating OA pathogenesis by influencing mitochondrial function (59). In inflammatory environments, mitochondria supply energy to chondrocytes while generating reactive oxygen species (ROS) (64). When ROS accumulation exceeds cellular antioxidant defense systems, oxidative stress (OS) ensues. Manganese superoxide dismutase (SOD2 or MnSOD), a subtype of superoxide dismutase (SOD), scavenges excess ROS within mitochondria. SIRT3 deacetylates SOD2, upregulating its activity and promoting mitochondrial redox balance. AMPK activation enhances mtDNA integrity and function in chondrocytes by increasing SOD2 activity via NAD-dependent SIRT3, thereby protecting cartilage from degradation (59, 63). SIRT1 (Sirtuin 1), another DNA-dependent histone deacetylase, acts downstream of the AMPK pathway. It inhibits P65’s transcriptional activity by deacetylating Lys310 (K310), thereby suppressing inflammatory responses (41). Therefore, disruption of the AMPK-SIRTs axis contributes to the onset and progression of OA.

#### 2.3.2 PI3K/Akt/mTOR signaling pathway

**Figure 1:**
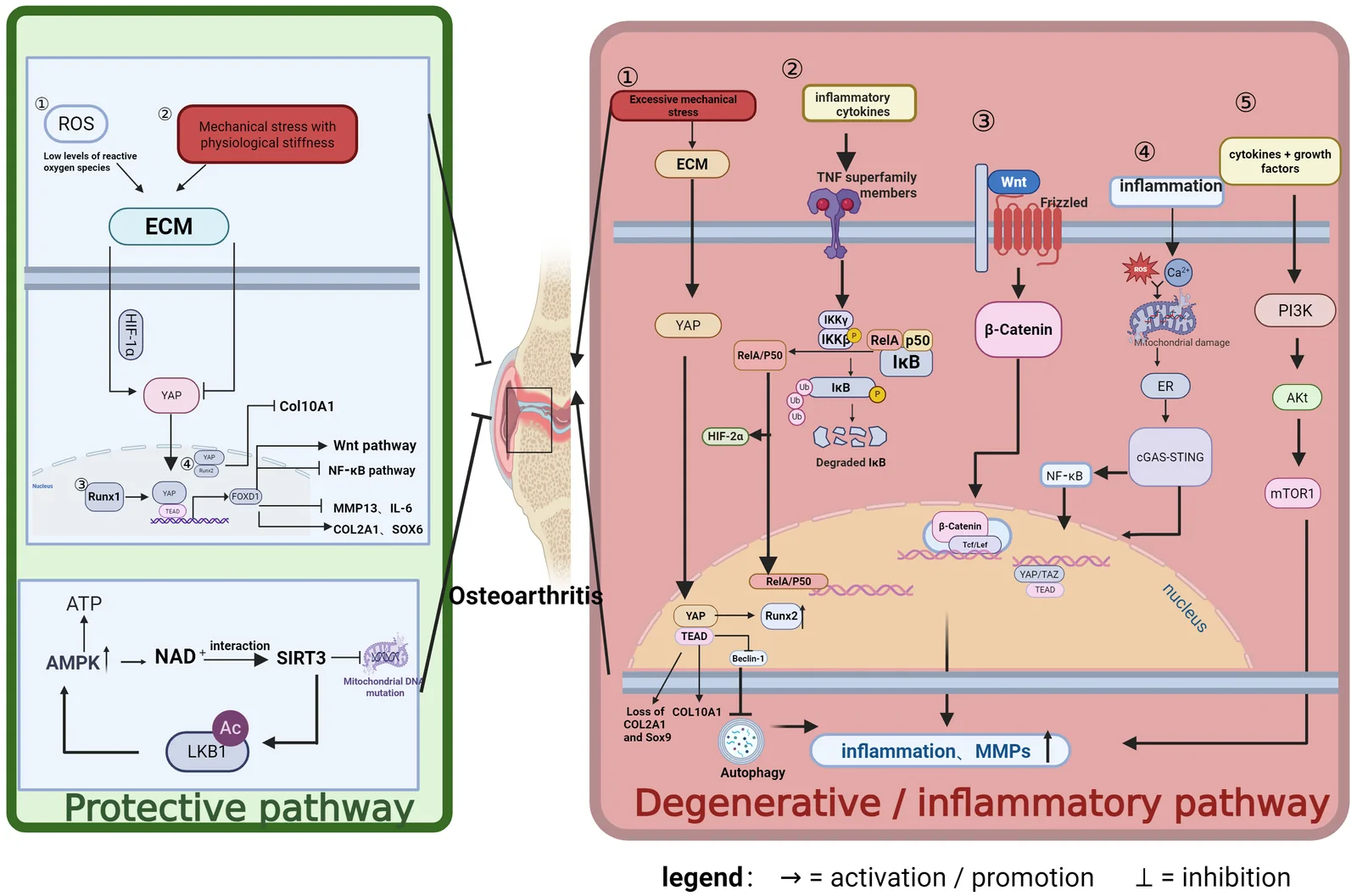
Signaling mechanism diagrams. This schematic diagram illustrates the protective and damaging mechanisms involved in signaling pathways associated with osteoarthritis. The left diagram depicts the protective mechanisms, whereas the right diagram portrays the damaging mechanisms. Numbers indicate key steps and molecular interactions, which are described in detail in the main text (see Section 2.1.1; 2.1.2; 2.2.1; 2.2.2; 2.3.1; 2.3.2). Created with BioRender.com.

The PI3K/Akt/mTOR signaling pathway exerts a significant influence on the pathogenesis of OA, primarily manifested in inflammatory responses during macrophage polarization, abnormal formation of subchondral bone, osteophyte formation, and mediation of chondrosynovial inflammation (65). Numerous cytokines and growth factors activate phosphoinositide 3-kinases (PI3Ks) through upstream signaling molecules such as protein kinase B (Akt), G protein-coupled receptors (GPCRs), and GTP-binding proteins. PI3Ks are categorized into three classes, among which Class I is the most classical and extensively studied. Upon activation, and then mediates the activation of downstream effector molecules, including Akt (also known as PKB) and mammalian target of rapamycin (mTOR), which are critically involved in cellular processes such as proliferation, survival, and apoptosis. The specific mechanism involves the recruitment of activated second messengers, such as PIP_3_, to the inner leaflet of the plasma membrane, where they facilitate the phosphorylation and activation of Akt by phosphoinositide-dependent kinase-1 (PDK1). This leads to the subsequent activation of the mTOR signaling pathway (66–68). The PI3K/Akt signaling pathway is crucial in modulating the anabolism and degradation of the ECM, with the equilibrium between these processes being vital for cartilage homeostasis. And the PI3K/AKT/mTOR axis is intimately involved in inflammatory signal transduction. Activation of this pathway enhances pro-inflammatory processes, thereby accelerating the progression of OA (69). In the synovium of OA patients, mTOR1 activation and increased M1 macrophages stimulate the production of inflammatory cytokines such as IL-1β, TNF-α, IL-6, and cyclooxygenase-2 (COX-2), inducing MMPs production that leads to chondrocyte destruction and degradation (70). Furthermore, PI3K/Akt activation via phosphorylation contributes to the activation of the NF-κB pathway, thereby mediating inflammatory responses (65, 71). mTOR functions as an inhibitor of autophagy, and its expression level is positively correlated with the production of pro-inflammatory cytokines (72). In post-traumatic OA patients, Akt phosphorylation promotes the proliferation of chondroblasts, leading to abnormal bone remodeling—specifically manifested as subchondral bone remodeling and osteophyte formation. Additionally, activation of mTOR complex 1 (mTORC1) induces bone sclerosis and enhances the secretion of CXCL12, which collectively contribute to the progression of OA. Nevertheless, chondrocyte proliferation is counterbalanced by concomitant cartilage degradation. Although PI3K/Akt/mTOR activation promotes osteochondral proliferation and bone formation, these effects are context-dependent and may exert dual roles in OA pathogenesis (73). Furthermore, this pathway is closely associated with synovial inflammation in OA (65). However, numerous studies have also demonstrated that Akt activation enhances the synthesis of type II collagen, promotes aggrecan accumulation, and upregulates Col2a1 expression, while simultaneously inhibiting the production of MMPs. Thereby, it helps maintain the balance between ECM synthesis and degradation (65, 74, 75). The precise mechanisms underlying these dual effects remain incompletely understood. The Akt pathway exerts a protective effect in osteoarthritis (OA) by modulating the NF-κB signaling pathway, which suppresses the production of pro-inflammatory cytokines and matrix metalloproteinases (MMPs). Additionally, it promotes chondrocyte survival and inhibits apoptosis (68). Furthermore, while PI3K/Akt activation has been shown to suppress MMP13 synthesis, its role in the catabolism of the cartilage ECM remains poorly elucidated (65). In summary, the PI3K/Akt/mTOR signaling pathway mediates diverse and complex pathological mechanisms in OA, exhibiting context-dependent chondroprotective or chondrodestructive effects. This underscores the pathway’s pivotal role in OA pathogenesis, although its detailed regulatory mechanisms require further investigation. Understanding how to maintain a balance between these opposing effects may offer novel therapeutic avenues for slowing OA progression. Schematic diagram of major signaling pathways transducing inflammatory mechanisms in OA as shown in **Figure 1**.

### 2.4 Other signaling pathways

Elevated expression and activation of MAPK signaling pathway components promote the development and exacerbation of OA. Specifically, in articular cartilage, p38—a member of the MAPK superfamily—undergoes phosphorylation and thereby activates the MAPK cascade. This in turn upregulates the expression of MMPs, promotes collagen degradation, accelerates cartilage destruction, and ultimately facilitates the development of OA (76). Activation of the RhoA/ROCK signaling pathway induces the reorganization of filamentous actin (F-actin), which compromises cellular traction forces (CTF) and disrupts chondrocyte homeostasis (77). This process reflects a spontaneous mechanical deterioration within the cell. Meanwhile, the JAK-STAT signaling pathway, activated by chemokines such as CXCL8 and CXCL11, enhances the expression of additional pro-inflammatory cytokines, thereby mediating inflammatory responses and promoting chondrocyte apoptosis (78). Subchondral bone remodeling is governed by the coupling of osteoblasts and osteoclasts. The OPG/RANKL/RANK signaling pathway regulates osteoclast activity and mediates cartilage degradation. Under normal mechanical stress, osteoblasts and chondrocytes secrete substantial amounts of RANKL, which binds to RANK on osteoclast precursors, promoting osteoclastogenesis. This process further activates inflammatory signaling pathways, such as NF-κB, leading to osteoclast maturation and subsequent cartilage degradation (79). The inducible ligand secreted by osteoblasts binds to RANKL to inhibit osteoclast differentiation and maturation, induce osteoclast apoptosis, suppress bone resorption, and result in abnormal remodeling of subchondral bone (80). This ultimately leads to the onset and progression of OA.

## 3 Ion channels: upstream regulators of mechanical–immune signaling in OA

Ion channels are transmembrane proteins that detect external stimuli and regulate cellular functions and activities. In recent years, growing evidence has highlighted their involvement in the pathogenesis of OA. These channels can mediate OA progression by triggering inflammatory responses and pain sensation in chondrocytes, thereby promoting cartilage degradation. Chondrocytes are highly differentiated cells whose plasma membranes contain a diverse array of ion channels and transporters, collectively referred to as the chondrocyte channelome (81). Various ion channels are implicated in the pathogenesis of OA. Transient receptor potential (TRP) channels, Piezo-type mechanosensitive ion channels (Piezo), and voltage-gated ion channels are activated under pathological conditions such as excessive mechanical stress, inflammatory stimuli, and disturbances in the articular cartilage microenvironment (4). This activation results in dysregulated sodium (Na^+^) and calcium (Ca²^+^) ion fluxes, which induce inflammatory pain and promote degradation of the chondrocyte extracellular matrix, thereby contributing to the development and progression of OA. Furthermore, other ion channels—including potassium (K^+^) channels, acid-sensing ion channels (ASICs), and chloride (Cl^-^) channels—have also been implicated in the pathogenesis of OA (2, 82, 83).

### 3.1 Mechanosensitive channels: direct mechanoreceptors of mechanical signals

As a weight-bearing tissue, articular cartilage is continuously subjected to various forms of mechanical stimuli, including compression, shear, and hydrostatic pressure. Piezo mechanosensitive ion channels, including Piezo1 and Piezo2, along with TRPV4 from the TRP channel family, are mechanosensitive channels that enable chondrocytes to detect changes in the mechanical microenvironment. The activation of Piezo channels is linked to intracellular Ca²^+^ release, which increases cell membrane tension and can lead to mechanical damage to the cartilage. Under conditions of mechanical stress overload, the activation of Piezo channels promotes Ca²^+^ influx, which damages chondrocyte mitochondria and can induce ferroptosis in chondrocytes, thereby contributing to osteoarthritis (OA) (84–86). Cell death is closely associated with the activation of the Piezo1 subtype, and studies have shown its involvement in cartilage degeneration (84, 85). Conversely, TRPV4 is activated under moderate mechanical stress, mediating physiological Ca²^+^ influx and regulating SOX9 expression and chondrocyte differentiation; however, its excessive activation can induce chondrocyte apoptosis and matrix degradation through mitochondrial dysfunction and Caspase-3-dependent pathways. In summary, the activation and opening of these ion channels trigger Ca²^+^ influx, converting mechanical signals into intracellular chemical signals, and serve as upstream receptors in the development of OA.

### 3.2 ATP/inflammation signaling pathways: TRPV/TRPA1 and P2X receptor channels

Transient Receptor Potential Ankyrin 1 (TRPA1) and TRPV1 play critical roles in mediating acute inflammation, degenerative changes, and joint pain in articular cartilage (86, 87). Both receptors regulate the release of the inflammatory cytokine IL-6 from chondrocytes (81, 88), which exacerbates inflammation, cartilage degeneration, and ECM degradation.P2X receptors are ATP-gated cation channels,P2X receptors are closely associated with chondrocyte degradation, inflammatory responses, and pain sensing. Among them, the P2X7 receptor (P2X7R) promotes channel opening upon sensing ATP energy changes, facilitating Na^+^ and Ca²^+^ influx (4, 89), thereby promoting chondrocyte pyroptosis (46), increased MMP-13 expression (90), and inflammatory induction (91). Studies indicate that P2X7R activation promotes IL-1β and IL-18 secretion, while P2X3 receptors (P2X3Rs) are associated with inflammatory pain transmission.

### 3.3 Acid sensors and electrolyte channels

Acid-sensing ion channels (ASICs) act as proton receptors that detect extracellular acidic signals. Upon activation of their expression, they contribute to the upregulation of inflammatory mediators, including interleukin-1β (IL-1β), interleukin-6 (IL-6), and tumor necrosis factor-α (TNF-α), as well as promote M1 macrophage polarization and interleukin-18 (IL-18) expression, thereby facilitating chondrocyte senescence (4). Moreover, cells sense extracellular acidification through proton-gated ASICs, leading to rapid and transient influxes of Na^+^ and Ca²^+^ (4, 92). The accumulation of inflammatory mediators within chondrocytes can further induce intracellular acidosis, which in turn activates acid-sensing ion channel 1a (ASIC1a) and exacerbates cartilage degradation. Notably, the ASIC3 subtype has been implicated in the development of chronic joint pain (4). In addition, chloride channels are involved in the pathogenesis of OA through multiple mechanisms, including alterations in the extracellular microenvironment, dysregulation of extracellular matrix metabolism, apoptosis, and pyroptosis (93). Kurita et al. reported that under hypotonic conditions in OA cartilage, the expression of the ClC-7 chloride channel is downregulated, leading to reduced acid-sensitive Cl^-^ currents and consequently aggravating chondrocyte apoptosis (94).

### 3.4 Voltage-gated and metabolism-associated ion channels: potassium and calcium channels

**Figure 2:**
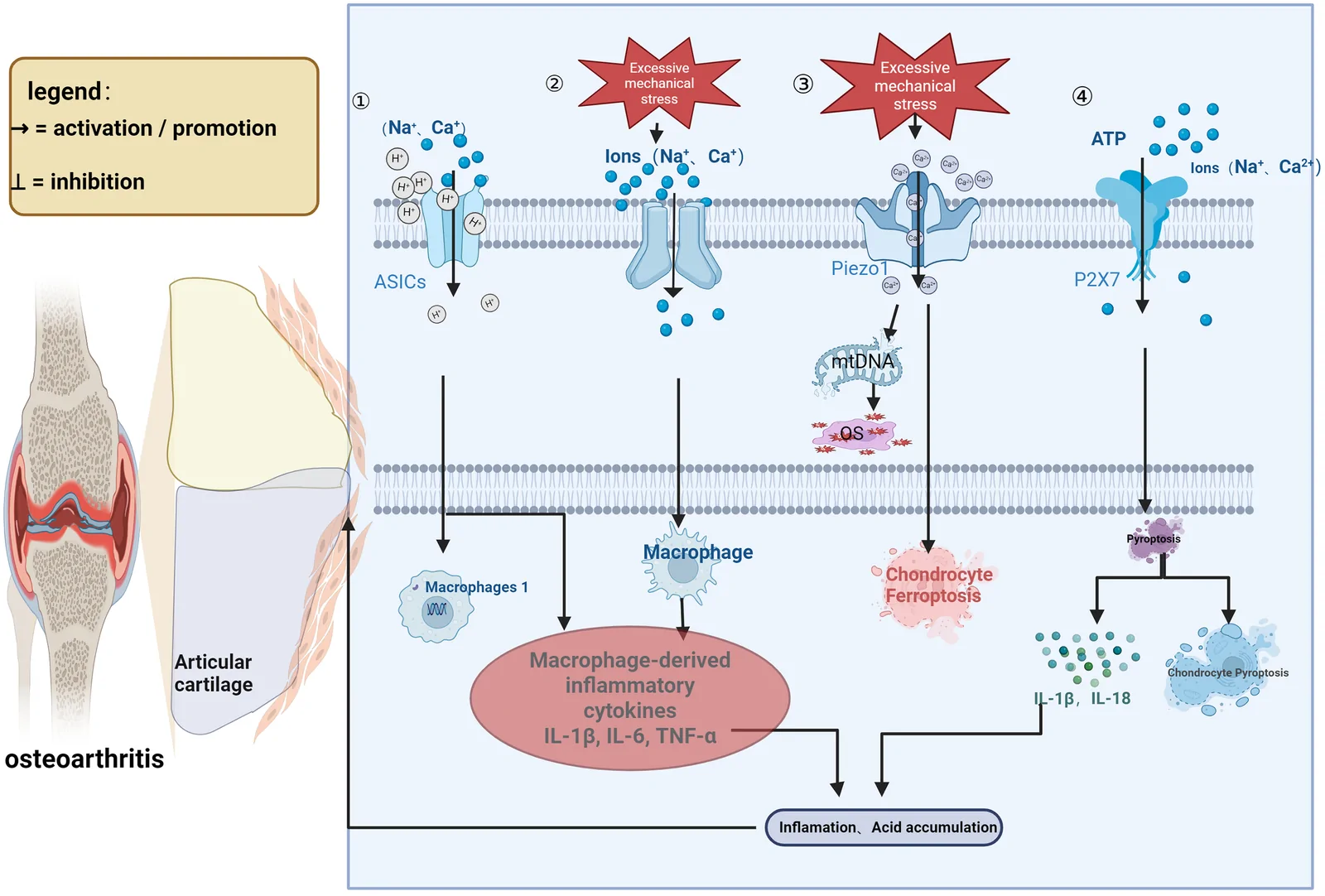
Schematic diagram of ion channel mechanisms in osteoarthritis pathogenesis. The numbered annotations correspond to various ion channels and their associated pathological processes, which are elaborated upon in the main text (refer to Section 3). The figure includes the following components: ① ASICs (acid-sensitive ion channels); ② TRPV1 (transient receptor potential vanilloid 1); ③ Piezo channels; ④ P2X7 receptors. Abbreviations used in the figure include: ASICs for acid-sensitive ion channels, TRPV1 for transient receptor potential vanilloid 1, P2X7R for P2X7 receptor, Na^+^ for sodium ion, Ca²^+^ for calcium ion, IL-1β for interleukin-1β, IL-6 for interleukin-6, IL-18 for interleukin-18, TNF-α for tumor necrosis factor alpha. Source: Created with BioRender.com.

The adenosine triphosphate (ATP)-sensitive potassium channel (KATP) in chondrocytes plays a key role in regulating chondrocyte metabolism and apoptosis, exerting diverse effects on the progression of OA. Experimental studies indicate that activation of ATP-sensitive potassium(KATP) channel can help maintain cartilage microenvironment homeostasis by modulating ERS and the extracellular signal-regulated kinase (ERK) pathway, thereby contributing to the preservation of cartilage integrity (95). Among these, voltage-gated potassium (Kv) channels are expressed in chondrocytes and contribute to various cellular functions. Abnormal expression of Kv channels is associated with ECM degradation and cartilage destruction (96, 97). Notably, downregulation of the long non-coding RNA KCNQ1 opposite strand/antisense transcript 1 (KCNQ1OT1), which is derived from the potassium voltage-gated channel subfamily Q member 1 (KCNQ1), promotes cartilage degradation and has been correlated with joint pain in OA (98). Voltage-gated calcium channels (Cav) play a crucial role in promoting inflammatory responses and matrix degradation through the mediation of Ca²^+^ influx. This influx significantly influences chondrocyte excitability, metabolic homeostasis, and various signal transduction processes. Moreover, the Ca²^+^ influx mediated by Piezo1 results in membrane depolarization, which subsequently activates Cav channels, leading to an extensive influx of Ca²^+^. This process triggers the activation of NF-κB and the NLRP3 inflammasome, thereby contributing to cartilage degeneration and the progression of OA (99–101). As shown in **Figure 2**.

The inflammatory mechanisms mediated by various ion channels in OA are illustrated in **Figure 2**.

## 4 The integrated immune–mechanical transduction network in OA

In addition to the degeneration of articular cartilage, OA is characterized by structural and functional abnormalities in the synovium and subchondral bone (102). The onset and progression of OA are primarily driven by immune responses that are mediated by mechanical signals, forming an immune-mechanical signaling network that regulates downstream signaling pathways (103, 104). Factors such as abnormal mechanical stress, changes in ECM stiffness, imbalances in intracellular and extracellular energy metabolism, and mitochondrial dysfunction lead to the activation of corresponding ion channels (99, 105). This activation subsequently triggers innate immune and cell stress-related signaling pathways, collectively driving the pathological progression of osteoarthritis (99). Abnormal mechanical stress in OA is manifested not only as overloading but also as underloading (106). Under conditions of excessive mechanical stress, Piezo1/2 and TRPV4 channels are activated, resulting in increased Ca²^+^ influx, which activates the NF-κB and MAPK signaling pathways, as well as YAP nuclear translocation, thereby promoting inflammatory responses and accelerating ECM degradation (107, 108). Conversely, insufficient mechanical loading deprives chondrocytes of physiological mechanical stimulation, weakening their ability to maintain cartilage homeostasis and promoting the degenerative progression of OA.

### 4.1 Mechanical and chemical signals as key upstream regulatory drivers

Mechanical and chemical stimuli, including mechanical stress, alterations in ECM stiffness, fluctuations in pH, inflammatory mediators, and the release of extracellular ATP, are significant upstream drivers of OA progression (99). Under conditions of mechanical overload, chondrocytes primarily perceive external stimuli through mechanosensitive ion channels, with Piezo1/2 and TRPV4 playing pivotal roles in mechanical transduction (109, 110). Additionally, damaged cartilage tissue can release damage-associated molecular patterns (DAMPs), such as mtDNA, fragments of degraded cartilage matrix, and ATP, which further exacerbate the inflammatory response (111). Concurrently, an acidic microenvironment, oxidative stress, and mitochondrial dysfunction also represent critical upstream factors in the pathological progression of OA (112, 113). Collectively, these changes activate ASIC1a, purinergic receptor channels (P2X7), TRP channels, and voltage-gated calcium channels (Cav), thereby establishing a vital signaling link between biochemical stress and the inflammatory response (99, 112).

### 4.2 Ion channels as upstream sensors of mechanical and chemical stimuli

In the pathogenesis of OA, cells respond to mechanical, chemical, and inflammatory signals by converting these stimuli into intracellular signaling events through upstream receptor ion channels. Under conditions of mechanical stress and abnormal ECM stiffness, the mechanosensitive ion channels Piezo1/2 and TRPV4 become activated, leading to an influx of Ca²^+^ (114). This influx subsequently regulates chondro-osteogenic remodeling, mitochondrial function, and the expression of inflammation-related genes (4, 109). Notably, inflammatory stimuli can further sensitize the mechanical responsiveness of Piezo1 in chondrocytes, enhancing their reaction to mechanical stimulation. This interaction amplifies inflammatory signals and promotes the upregulation of Piezo1 expression (85), thereby creating a positive feedback loop between inflammation and mechanical injury.

The P2X7 receptor is activated upon sensing ATP released during cellular damage, which triggers the influx of Na^+^ and Ca²^+^ ions, mediating inflammatory responses and pain, and promoting pyroptosis and ECM degradation (46, 115). TRP channels, ASIC1a, potassium (K^+^) channels, chloride (Cl^-^) channels, and voltage-gated channels collectively contribute to the onset and progression of OA by regulating Ca²^+^ influx, maintaining membrane potential homeostasis, activating inflammatory signals, and producing ROS. Ion channels are not isolated components of OA; rather, they function as upstream receptors in the pathogenesis of OA, acting as a hub that integrates mechanical stress, extracellular chemical signals, and immune signaling pathways into downstream signaling cascades.

### 4.3 Signal transduction linking ionic signals to immune responses

Upon activation of ion channels, the transmembrane flow of ions such as Ca²^+^ and Na^+^ creates a critical intermediary layer that transmits extracellular signals into the cell, thereby triggering an immune response. The Piezo1 and TRPA1 channels mediate Ca²^+^ influx, initiating downstream signaling pathways such as NF-κB and MAPK, which are dependent on Ca²^+^. These pathways promote the secretion of inflammatory factors and the production of MMPs, as well as the activation of the NLRP3 inflammasome. Additionally, oxidative stress exacerbates the degradation of the cartilage matrix and induces pyroptosis. In a study involving rats with OA using a destabilization of the medial meniscus (DMM) surgical model (116), excessive mechanical stress led to the activation of Piezo1, which mediates the downstream PI3K/AKT/mTORC1 pathway, thereby promoting ECM degradation. The P2X7 receptor mediates the downstream AMPK/mTOR axis, regulating the balance between pyroptosis and autophagy, with the outcome being dependent on the degree of its activation. Moderate activation of P2X7 has a protective effect on cartilage, while excessive activation results in NLRP3 inflammasome activation, Caspase-1 cleavage, and the release of inflammatory factors such as IL-1β, which promote cartilage pyroptosis and degradation (91). Furthermore, P2X7 exacerbates inflammation and pyroptosis through crosstalk with the NF-κB/NLRP3 pathway (46).

Ca²^+^ overload and sustained ROS production lead to mitochondrial damage and the release of mtDNA, which activates the cGAS-STING innate immune pathway. This activation promotes inflammatory amplification and facilitates the release of mtDNA into the cytoplasm (49). Excessive activation of STING triggers the release of pro-inflammatory cytokines such as IL-6, TNF-α, and IL-1β via the NF-κB pathway, which serves as an activation signal for the NLRP3 inflammasome. Consequently, the cGAS-STING pathway and the NLRP3 inflammasome share upstream mtDNA/ROS signaling, exhibiting positive-feedback cross-activation (117–119). Furthermore, the cGAS-STING pathway, in conjunction with NF-κB, enhances the production of MMPs and ADAMTs, activating the RANK/RANKL pathway and leading to cartilage matrix degradation (117). Currently, ferroptosis is also recognized as playing a significant role in the onset and progression of OA. Under conditions of mechanical stress overload, Piezo1 mediates Ca²^+^ influx, inducing mitochondrial dysfunction and ROS accumulation, which promotes lipid peroxidation and inhibits glutathione peroxidase 4 (GPX4) activity, ultimately leading to cellular ferroptosis. This process is closely associated with the AMPK/SIRT mitochondrial protective pathway (100, 120, 121).

### 4.4 Convergence and signal amplification in signaling pathways

Ion channels convert mechanical and inflammatory stimuli into intracellular chemical signals, which do not operate in isolation but rather form a regulatory network characterized by cascading amplification through cross-talk. Among these, NF-κB functions as the central transcriptional hub, integrating Piezo-1-mediated Ca²^+^ influx, ROS production, ERS, and P2X7 receptor-mediated inflammatory signals. This integration collectively drives the transcriptional expression of MMPs and various inflammatory factors, while simultaneously upregulating NLRP3 inflammasome-associated molecules (85). Under stimuli such as intracellular K^+^ efflux and mitochondrial stress, the NLRP3 inflammasome is activated, promoting the release of IL-1β. This release, through positive feedback, further activates the NF-κB signaling pathway, mediating the amplification of inflammation and the process of pyroptosis (122, 123). Concurrently, the cGAS–STING signaling pathway connects mtDNA leakage to the activation of the innate immune response, synergistically enhancing the intensity and persistence of the inflammatory response. Furthermore, under the influence of abnormal ECM stiffness and mechanical stress, the Hippo/YAP and Wnt/β-catenin signaling pathways exert transcriptional regulatory functions within the cell nucleus, participating in the regulation of chondrocyte hypertrophy, proliferation, matrix remodeling, and chondral degeneration. In OA, the inhibitory effect of YAP on the TAK1–NF-κB pathway is diminished, further amplifying the inflammatory signaling cascade (26, 124). AMPK-SIRT3 and the PI3K/AKT/mTOR pathways play crucial roles in regulating cellular processes by modulating mitochondrial quality control, autophagy, and energy metabolism. During the early stages of OA or under mild mechanical stress, AMPK is activated, which helps maintain MQC and facilitates adaptive cartilage repair. However, within an inflammation-amplified network, the PI3K/AKT/mTOR pathway exacerbates oxidative stress by inhibiting autophagy, thereby promoting degenerative damage to cartilage. Furthermore, necroptosis is involved in amplifying inflammation in OA. It can be initiated by TNF-α/NF-κB-related inflammatory pathways and executed via the RIPK1/RIPK3/MLKL axis, ultimately leading to cell lysis and the release of significant amounts of pro-inflammatory substances, which further amplify the inflammatory response (125, 126). Necroptosis releases DAMPs that activate the NLRP3 inflammasome, resulting in the production of large quantities of IL-1β (127), which subsequently activates neighboring macrophages and synovial fibroblasts, prompting them to produce and release substantial amounts of TNF-α, thereby exacerbating necroptosis (128). Concurrently, programmed cell death releases mtDNA into the cytoplasm, activating the cGAS-STING pathway, which triggers the release of large amounts of TNF-α via the NF-κB pathway, further exacerbating programmed cell death (117, 129). Additionally, the activated STING pathway can induce the expression of type I interferon (IFN-I) as well as various chemokines such as CXCL10 and CCL5 (130). These factors strongly recruit macrophages and T cells to infiltrate the local inflammatory site, where they release large amounts of TNF-α and ROS, thereby expanding the inflammatory network (131–133).

## 5 Context-dependent bidirectional regulation of the Hippo/Yap and PI3K/Akt/mTOR signaling pathways

### 5.1 Bidirectional regulatory mechanisms of the hippo/YAP signaling pathway

The Hippo/YAP signaling pathway plays a bidirectional regulatory role in OA. In a soft matrix environment, YAP remains predominantly localized in the cytoplasm or enters the nucleus at low levels in a dynamic manner, promoting the expression of COL2A1 and inhibiting abnormal chondrohypertrophy, thus exerting a chondroprotective effect (15, 26). However, when mechanical stress becomes excessive, YAP undergoes sustained nuclear translocation and binds to the TEAD transcription factor, which induces the upregulation of RUNX2 and COL10A1 expression. This process promotes the hypertrophic differentiation of chondrocytes, subchondral bone resorption, and calcification, accompanied by a decrease in SOX9 expression (15, 23), thereby driving the progression of OA. YAP nuclear translocation exhibits marked context-dependence within the Hippo/YAP pathway, with its effects varying according to the cellular environment, the type of mechanical stimulus, and the stage of the disease, suggesting that clinical interventions should be tailored to specific stages.

### 5.2 Bidirectional regulatory mechanisms of the PI3K/Akt/mTOR signaling pathway

The PI3K/Akt/mTOR signaling pathway exerts distinct, tissue-specific roles across different regions of the joint, reflecting its functional heterogeneity in OA. In articular cartilage, Akt activation is generally considered to promote anabolic processes and exert anti-apoptotic effects. Related studies have demonstrated that activation of the PI3K/Akt pathway enhances the synthesis of type II collagen and aggrecan in intervertebral disc cells, thereby conferring a chondroprotective effect (134, 135). In contrast, within the synovium, Akt activation concurrently suppresses autophagy via mTORC1 activation, promotes M1 macrophage polarization, and facilitates the release of pro-inflammatory cytokines such as IL-1β and TNF-α, thereby exacerbating synovial inflammation (136, 137). In subchondral bone, Akt signaling contributes to excessive osteoblast differentiation and aberrant bone remodeling, ultimately leading to osteophyte formation and subchondral bone sclerosis (138). In summary, the net effect of this pathway is determined by the dynamic balance between Akt activation and mTORC1-mediated autophagy inhibition as well as inflammatory amplification across different joint tissues. (The context-dependent dual roles of these pathways are summarized in **Table 1**).

**Table 1:** Context-dependent dual roles of Hippo/YAP and PI3K/Akt/mTOR signaling pathways in OA.

Signaling pathway	Cell type/tissue	Mechanical/microenvironmental conditions	Activation level/localisation	Effect (protective vs detrimental)	Key molecular mechanisms
Hippo/YAP	Chondrocyte	Soft ECM	Cytoplasmic retention/Low-level nuclear translocation	Protective: promotes COL2A1 expression, inhibits chondrocyte hypertrophy	SOX9↑, RUNX2↓
Hippo/YAP	Chondrocyte	Mechanical overloading	Sustained nuclear translocation	Detrimental: promotes chondrocyte hypertrophy, calcification, and ECM degradation	TEAD↑, RUNX2↑, COL10A1↑, SOX9↓
PI3K/Akt/mTOR	Cartilage	/	Moderate Akt activation	Protective: promotes anabolic effects and inhibits apoptosis	Col II↑, Aggrecan↑
PI3K/Akt/mTOR	Synovium	/	mTORC1 activation	Protective: promotes anabolic effects, inhibits apoptosis	autophagy↓, IL-1β↑, TNF-α↑
PI3K/Akt/mTOR	Subchondral bone	/	Sustained Akt/mTOR activation	Detrimental: promotes bone remodeling and osteophyte formation	Osteoblast differentiation↑

↑, increased expression or activation; ↓, decreased expression or inhibition.

## 6 Conclusions

The prevalence of OA is increasing annually and represents a growing global health challenge. Current medical interventions are unable to fully reverse OA and are limited to managing symptoms and delaying disease progression. Therefore, the development of effective clinical treatments remains imperative. The pathogenesis of OA is multifactorial and highly complex, with inflammatory responses and osteochondral degradation representing central processes in disease advancement. With advances in research, increasing attention has been devoted to the roles of cellular signaling pathways and ion channels in the pathogenesis of OA. It has been recognized that ion channels, upon sensing extracellular stimuli, mediate alterations in ion flux, which are subsequently transduced into intracellular signals. These signals, in turn, regulate multiple signaling pathways that contribute to the initiation and progression of OA. The Hippo/YAP signaling pathway and the PI3K/Akt/mTOR signaling pathway play bidirectional regulatory roles in OA development. They are involved in maintaining cartilage homeostasis and facilitating repair processes; however, they may also promote degeneration under conditions of excessive mechanical stress or within an inflammatory microenvironment. The NF-κB signaling pathway and the cGAS-STING signaling pathway mediate the degradation of the ECM in chondrocytes by modulating inflammatory responses. The Wnt/β-catenin signaling pathway primarily induces OA by activating the production of MMPs and ADAMTs. Meanwhile, the AMPK/SIRT axis plays a crucial protective role in energy metabolism and mitochondrial homeostasis, and its imbalance is regarded as a significant metabolic basis for the degenerative progression of OA. Ion channels function as upstream receptors for mechanical and chemical signals in the progression of OA. Channels such as Piezo, TRP, P2X, and ASIC convert external mechanical stress and inflammatory stimuli—including acidity—into intracellular signals. Among these, the roles of Piezo and TRPV4 have garnered significant attention in OA research; they further connect to inflammatory networks such as NF-κB, MAPK, and NLRP3, thereby driving cartilage degeneration and the development of pain. The transformed intracellular signals link ion channels to signaling pathways, forming complex interactions that constitute a mechanosensory-inflammatory-immune network, which collectively regulates the onset and progression of OA. Consequently, interventions targeting a single pathway often fail to completely halt disease progression; future strategies may necessitate the coordinated regulation of multiple pathways. At the clinical level, the signaling pathway–ion channel networks summarized in this review provide a theoretical foundation for OA interventions. For example, targeting key inflammatory signaling nodes such as NF-κB/NLRP3 and cGAS-STING may help alleviate synovial inflammation and pain; modulating mechanotransduction pathways (such as Piezo/TRPV4) may enhance chondrocytes’ ability to adapt to abnormal mechanical environments; and activating the AMPK-SIRT axis may delay cartilage degeneration at the metabolic level. These findings offer new insights for exploring “mechanical-immune integrated” therapeutic strategies. However, our current understanding of the OA signaling pathways is still primarily based on *in vitro* cell experiments and animal models. The spatial heterogeneity and temporal dynamics of these pathways within human joints remain unclear. Furthermore, the upstream-downstream relationships between different pathways—particularly the transition between inflammatory amplification and protective responses—remain controversial and require further research to provide insights for targeted therapy at specific stages of OA.

## Glossary

OA: Osteoarthritis
NSAIDs: non-steroidal anti-inflammatory drugs
Hippo/YAP: the Hippo/Yes-associated protein
NF-κB: the nuclear factor kappa B
cGAS–STING: cyclic GMP–AMP synthase–stimulator of interferon genes
AMPK: the adenosine monophosphate-activated protein kinase
PI3K/AKT/mTOR: phosphoinositide 3-kinase/protein kinase B/mammalian target of rapamycin
ECM: extracellular matrix
HIF-1α: hypoxia-inducible factor-1 Alpha
FOXD1: Forkhead Box D1
TEADs: TEA domain transcription factors
MSCs: mesenchymal stem cells
COL2A1: Collagen Type II Alpha 1 Chain
Tcf/Lef: T cell factor/lymphoid enhancer factor
PKC: protein kinase C
WISP-1: Wnt1- inducible signaling pathway protein 1
JNK: c-Jun N-terminal kinase
TNFα: Tumor Necrosis Factor α
NO: nitric oxide
iNOS: inducible nitric oxide synthase
PGE₂: prostaglandin E₂
HIF-2α: hypoxia-inducible factor 2α
ADAMTS: A Disintegrin and Metalloproteinase with Thrombospondin Motifs
TGF-β: transforming growth factor-β
TAK1: transforming growth factor-β-activated kinase 1
MQC: mitochondrial quality control
OS: oxidative stress
NAD⁺: nicotinamide adenine dinucleotide
LKB1: liver kinase B1
mtDNA: mitochondrial DNA
AMPK-SIRT3 PFL: AMP-activated protein kinase (AMPK)–sirtuin 3 (SIRT3)–pyruvate formate-lyase (PFL) axis
ROS: reactive oxygen species
IRF3: interferon regulatory factor 3
TBK1: tank-binding kinase 1
IKK: IκB kinase
TLR-2: Toll-like receptor 2
NOD2: nucleotide-binding oligomerization domain-containing protein 2
GPCRs: G protein-coupled receptors
PDK1: phosphoinositide-dependent kinase-1
COX-2: cyclooxygenase-2
MMPs: matrix metalloproteinases
F-actin: filamentous actin
TRP: transient receptor potential (TRP) channels
Piezo: Piezo mechanosensitive ion channels
TRPA1: transient receptor potential vanilloid 1
ASIC1a: acid-sensing ion channels
KATP: ATP-sensitive potassium channel
ERK: extracellular signal-regulated kinase
KCNQ1OT1: KCNQ1 opposite strand/antisense transcript 1
KCNQ1: potassium voltage-gated channel subfamily Q member 1
P2X7R: P2X7 receptor
P2X3Rs: P2X3 receptors
SOD2 or MnSOD: manganese superoxide dismutase
